# Electronic screen device usage and screen time among preschool-attending children in a suburban area of Sri Lanka

**DOI:** 10.1186/s12887-022-03452-6

**Published:** 2022-07-04

**Authors:** Asanka Rathnasiri, Harsha Rathnayaka, Nirmani Yasara, Sachith Mettananda

**Affiliations:** 1 University Paediatrics Unit, Colombo North Teaching Hospital, Ragama, Sri Lanka; 2 Department of Paediatrics, Faculty of Medicine, University of Kelaniya, Thalagolla Road, Ragama, 11010 Sri Lanka

**Keywords:** Screen time, Preschool children, Television use, Screen device

## Abstract

**Background:**

Excessive use of screen devices and screen time are increasing health problems in children. We aim to describe the electronic screen device usage and determine the factors associated with their use among preschool-attending children in a suburban population in Sri Lanka.

**Methods:**

A cross-sectional study was conducted in a suburban Medical Officer of Health area of Sri Lanka from January to March 2020. All children aged between 36–59 months attending ten randomly selected preschools were recruited. Data were collected using a parent-administered questionnaire and analysed using binary logistic regression in SPSS. The prevalence of electronic device usage, the average time spent on each device, and factors associated with individual device usage were analysed.

**Results:**

A total of 340 children (Male-48%; mean age-50.1 ± 6.9 months) were recruited. Electronic devices were used by 96% of children. The most common devices were the television (87%) and the smartphone (63%). Of the children who used electronic devices, 60% exceeded the recommended screen time limit of one hour per day, 21% used devices for more than two hours per day, and 51% commenced using devices by two years of age. The higher education level of the father was independently associated with the use of smartphones and laptops and daily screen time of more than one hour (*p* < 0.05 for all). Male sex and being the only child were significantly associated with the use of smartphones, whereas maternal employment was associated with the use of laptops (*p* < 0.05 for all).

**Conclusions:**

Electronic screen devices were used by 96% of preschool-attending children, and over 60% used them for more than the recommended daily upper limit of one hour. Higher paternal education, maternal employment and being the only child were significantly associated with electronic screen device use.

## Background

Excessive use of screen devices in children is associated with several health hazards. These include a negative impact on language development, cognition, social interactions, sleep, learning, behaviour and non-communicable diseases [1–3]. Several guidelines and recommendations were developed in recent years to set limits for screen device use by children. The World Health Organisation recommends that children of 3–4 years limit screen time to less than 60 min per day [4]. The most recent guideline issued in 2016 by the American Academy of Paediatricians also recommends a maximum of one hour of screen time per day in children aged between 2–5 years [5]. A number of recent studies done in the Americas and Europe have evaluated screen time among pre-school children in high-income countries against these guidelines [6]. However, studies evaluating the conformity of preschool children in low- and middle-income countries to these guidelines are sparse [7].

Increased use of electronic screen devices has become a significant health problem in the paediatric population worldwide [8]. Studies done in developed countries have shown a rapid increase in the rate of screen device usage among children over the past decade [9, 10]. Similarly, the average age of using electronic screen devices has declined steadily [11]. Of note, the American Academy of Paediatrics stated that screen time is the most common wake time activity in many children and in some, it was longer than their sleep time [12].

Sri Lanka is a lower middle-income country in South Asia that has a population of 21.5 million (2021 data). Compared to other developing countries, the literacy rates of both genders are high in Sri Lanka. Due to this, the electronic device usage by the Sri Lankan population is higher than that of other countries in the region. A survey done in 2020 revealed 31.8 million mobile phone connections in Sri Lanka, which corresponds to 1.38 connections per person [13].

Parallel to the rise in screen media use in adults, it is expected that screen device use and related health adversities in children have also risen. Recent studies have reported associations between high screen time and migraine and behavioural problems among older children in Sri Lanka [14, 15]. However, the conformity to international recommendations on screen time among preschool children in Sri Lanka has not been studied. This study describes the electronic device usage and screen time and determines the factors associated with their use among preschool-attending children in a suburban population of Sri Lanka.

## Methods

This cross-sectional study was conducted in the Homagama Medical Officer of Health division in the Western province of Sri Lanka. Homagama is a semi-urban suburb located 25 km away from Colombo, the commercial capital of Sri Lanka. It has a population density of 2,011/km^2^, and it classically represents the suburban communities of Sri Lanka [16]. This study was conducted from January to March 2020, before the emergence of the COVID-19 pandemic in Sri Lanka. It was conducted in accordance with the regulations of the Declaration of Helsinki, and ethical approval was obtained from the Ethics Review Committee of the Sri Lanka College of Paediatricians. Administrative approvals were obtained from the Regional Director of Health Services and the Medical Officer of Health of the area.

Ten preschools in the area were randomly selected after obtaining permission from the head teachers. A complete list of preschools in the area was obtained from the Medical Officer of Health, and ten preschools were selected randomly using a random number table. Preschools in Sri Lanka provide early childhood education for children aged between 3–5 years and are regulated by local government authorities. They are privately owned and institution-based, routinely function from 8 am to 12 noon on weekdays and do not provide meals or allow screen time for children. All children aged between 36 to 59 months attending the selected preschools were recruited after obtaining informed written consent from a parent or a guardian. Children with developmental delay, autism spectrum disorder, special educational needs and visual or hearing impairment were excluded from the study. The sample size was calculated based on a 27% anticipated prevalence rate for screen time exceeding recommendations as reported in a previous study from the region [17]. The calculated minimum sample size for a 95% desired precision was 303. We decided to recruit a minimum of 334 after allowing for a 10% nonresponse rate.

Data were collected using a pre-tested, parent-administered questionnaire available in Sinhala, Tamil or English as preferred by the participants. The questionnaires and participant information sheets were distributed to the parents of selected preschools by investigators, and completed questionnaires were collected on the same day. The questionnaire contained questions to assess the socio-demographic characteristics, availability and usage of electronic screen devices, the time child spent on each device and parental perception of screen device usage. Data on the availability and usage of electronic devices were gathered using separate questions to be answered ‘yes’ or ‘no’ for individual devices- television, smartphone, tablet, desktop, laptop and others. Data on time spent on each device was gathered as the average time per day for each device to be filled by the parent. The age at first usage was captured by a question with a single choice to be selected from multiple choices of predetermined age ranges. Data on types of programmes viewed were gathered using questions to be answered ‘yes’ or ‘no’ for cartoons, tele dramas, movies, educational programmes, and nursery rhymes. An ‘other’ option to fill in the blanks was included to gather data on other types of programmes viewed. Data on parental perception was gathered from structured questions to be answered ‘yes’ or ‘no’ except for the question “What measures have you taken to limit the use of screen devise usage by your child?” which was kept blank for parents to fill.

Data were analysed using IBM SPSS statistic 25.0. Categorical data are expressed as frequencies and percentages. Binary logistic regression was used to determine associations between categorical variables, and adjusted odds ratios were presented. In the logistic regression models that examined the independent associations for individual device use, gender, age group, maternal and paternal education level, maternal employment status, number of children in the family and monthly family income were included as covariates. The cut-off for statistical significance was set at *p* < 0.05.

## Results

A total of 340 children were recruited for the study. In 252 (74.1%) of them, the informant was mother, whereas, in 83 (24.4%) and 5 (1.5%) participants, the informants were father and guardian, respectively. The mean age of the study population was 50.1(± 6.9) months, and general characteristics are summarised in Table 1.

**Table 1:** General characteristics of the study population

**Characteristic**	**Frequency** **(*N* = 340)**	**Percentage**
Sex of the child^a^		
Male	154	48.1
Female	166	51.9
Age of the child		
3 – 4 years	139	40.9
4 – 5 years	201	59.1
Age of the mother		
< 20 years	5	1.5
21–30 years	71	20.9
31–40 years	240	70.6
> 40 years	24	7.1
Education level of the mother^b^		
Primary education	1	0.3
Secondary education (Ordinary Level)	52	15.4
Secondary education (Advance Level)	160	47.3
Higher education	125	36.9
Employment status of the mother^c^		
Unemployed	165	49.3
Employed	170	50.7
Age of the father		
< 20 years	1	0.3
21–30 years	33	9.7
31–40 years	241	70.8
> 40 years	65	19.1
Education level of the father^d^		
Primary education	6	1.8
Secondary education (Ordinary Level)	93	28.1
Secondary education (Advance Level)	139	42.0
Higher education	93	28.1
Number of children in the family		
One	139	40.9
Two	156	45.9
Three	42	12.4
Four	3	0.9
Monthly family income^e^		
< LKR 25,000	19	5.7
LKR 25,000–50,000	129	38.9
LKR 50,000–100,000	127	38.3
> LKR 100,000	57	17.2
Age of commencement of using electronic screen devices		
Less than 6 months	3	0.9
7 – 12 months	56	16.5
13 – 24 months	116	34.9
25 – 36 months	89	26.8
37 – 48 months	58	17.5
49 – 59 months	9	2.7

Data missing from:

^a^20 subjects

^b^2 subjects

^c^5 subjects

^d^9 subjects

^e^8 subjects.; USD 1 = LKR 200

### Availability and usage of screen devices

The common screen devices available in households were televisions [320 (94.1%)], smartphones [308 (90.1%)] and laptops [141 (41.5%)]. Tablet devices and desktop computers were available in 56 (16.5%) and 57 (16.8%) households, respectively. The usage of the screen devices by children showed a distribution parallel to the availability. The television was the most commonly used screen device which was used by 295 (86.8%) preschool children, followed by smartphones [214 (62.9%)] and laptops [54 (15.9%)]. Tablet devices and desktop computers were used by 28 (8.2%) and 19 (5.6%) children, respectively. The most commonly viewed programmes were cartoons [256 (75.3%)] and nursery rhymes [214 (62.9%)]. A majority [175 (52.3%)] of children had started using screen devices before 24 months of age, of which 59 (17.4%) were exposed to screen devices even before the first birthday (Table 1).

### Screen time

Only 14 (4.1%) children were not having any screen time at all, whereas 205 (60.6%) had screen time exceeding one hour per day. Seventy-one (21.1%) reported more than two hours of screen time per day (Table 2). Of that, nine (2.7%) children used screens for more than four hours per day. Most time was spent watching television and smartphones with 83 (24.5%) and 25 (7.3%) children watching them for over one hour per day.

**Table 2:** Distribution of children by daily screen time and devices [*N* = 339](%)

Screen time per day	Type of the screen devices					
	**Total**	**Television**	**Smartphone**	**Tablet**	**Desktop**	**Laptop**
None	14 (4.1)	47 (13.9)	131 (38.6)	314 (92.6)	320 (94.4)	299 (88.2)
< 30 min	40 (11.8)	92 (27.1)	140 (41.3)	15 (4.4)	12 (3.5)	27 (8.0)
31 min – 1 h	80 (23.6)	117 (34.5)	43 (12.7)	8 (2.3)	3 (0.9)	9 (2.7)
1–2 h	134 (39.5)	73 (21.5)	12 (3.5)	1 (0.3)	2 (0.6)	2 (0.6)
2–3 h	46 (13.6)	7 (2.1)	12 (3.5)	1 (0.3)	2 (0.6)	2 (0.6)
3–4 h	16 (4.8)	2 (0.6)	1 (0.3)	0	0	0
> 4 h	9 (2.7)	1 (0.3)	0	0	0	0

### Caregiver perceptions and practices on screen device usage

Of the caregivers, 251 (75.4%) thought that screen devices are helpful for their child's learning. However, 52 (15.3%) caregivers perceived that their children are addicted to screen devices. Only 254 (76.3%) caregivers had taken steps to limit screen time and device usage. The most commonly used strategies were encouraging alternate activities (48.8%) and limit setting (47.2%). Educating the children on the possible harms of screen use was adopted by 3.8% of the parents.

### Factors associated with screen time exceeding one hour per day

Of the children who used screen devices for more than one hour per day, 90 (48.9%) were males, and 94 (51.1%) were females (Table 3). The higher education level of the father was independently associated with the screen time of more than one hour per day (*p* < 0.01).

**Table 3:** Factors associated with screen time exceeding one hour per day ^a^

**Socio-demographic factor**	**Number (%) with screen time > 1 h per day**	**Adjusted Odds Ratio (95% CI)**	***P*-value**
Gender of the child			
Male (*N* = 148)	90 (60.8%)	0.99 (0.61–1.60)	0.97
Female (*N* = 153)	94 (61.4%)		
Age of the child			
4–5 years (*N* = 178)	113 (63.5%)	1.34 (0.82–2.17)	0.23
3–4 years (*N* = 123)	71 (57.7%)		
Mother's education level			
> Ordinary level (*N* = 255)	159 (62.4%)	1.28 (0.19–1.28)	0.51
≤ Ordinary level (*N* = 46)	25 (54.3%)		
Father's education level			
> Ordinary level (*N* = 207)	140 (67.6%)	2.65 (1.45–4.84)	< 0.01
≤ Ordinary level (*N* = 94)	44 (46.8%)		
Mother's employment status			
Employed (*N* = 149)	99 (66.4%)	1.28 (0.76–2.14)	0.33
Unemployed (*N* = 152)	85 (55.9%)		
Number of children			
Only child (*N* = 126)	82 (65.1%)	1.30 (0.79–2.13)	0.29
More than one child (*N* = 175)	102 (58.3%)		
Monthly family income			
> LKR 50,000 (*N* = 168)	106 (63.1%)	1.15 (0.67–1.96)	0.60
≤ LKR 50,000 (*N* = 133)	78 (58.6%)		

^a^The analysis was done using binary logistic regression. 301 subjects with completed socio-demographic data were included in the analysis. Adjusted odds ratios were determined by adjusting to all other variables in the table

### Factors associated with electronic screen device usage

Finally, we looked at the factors associated with individual screen device usage (Table 4). The higher education level of the father was independently associated with the use of smartphones (*p* < 0.001) and laptops (*p* < 0.01). Male sex (*p* < 0.05) and being the only child (*p* < 0.01) was associated with the use of smartphones, whereas maternal employment was associated with the use of laptops (*p* < 0.05).

**Table 4:** Factors associated with television, smartphone and laptop use of study population^a^

Socio-demographic factor	Television users			Smartphone users			Laptop users		
	**Number (%)**	**AOR** **(95% CI)**	***P***-value	**Number (%)**	**AOR** **(95% CI)**	***P*-value**	**Number (%)**	**AOR** **(95% CI)**	***P-value***
Gender of the child									
Male (*N* = 148)	127 (85.5%)	0.77 (0.38–1.56)	0.47	102 (68.9%)	1.70 (1.02–2.83)	< 0.05	25 (16.9%)	1.34 (0.68–2.62)	0.39
Female (*N* = 153)	136 (88.9%)			89 (58.2%)			21 (13.7%)		
Age of the child									
4–5 years (*N* = 178)	164 (92.1%)	2.84 (1.39–5.82)	< 0.01	108 (60.7%)	0.76 (0.46–1.28)	0.31	28 (15.7%)	1.14 (0.58–2.25)	0.69
3–4 years (*N* = 123)	99 (80.5%)			83 (67.5%)			18 (14.6%)		
Mother's education level									
> Ordinary level (*N* = 255)	223 (87.5%)	1.06 (0.34–3.25)	0.91	167 (65.5%)	0.76 (0.35–1.66)	0.50	43 (16.9%)	0.71 (0.16–2.98)	0.63
≤ Ordinary level (*N* = 46)	40 (87.0%)			24 (52.2%)			3 (6.5%)		
Father's education level									
> Ordinary level (*N* = 207)	180 (87.0%)	0.64 (0.25–1.63)	0.35	149 (72.0%)	3.39 (1.81–6.33)	< 0.001	43 (20.8%)	6.67 (1.75–25.3)	< 0.01
≤ Ordinary level (*N* = 94)	83 (88.3%)			42 (44.7%)			3 (3.2%)		
Mother's employment status									
Employed (*N* = 149)	133 (89.3%)	1.26 (0.59–2.65)	0.54	107 (71.8%)	1.59 (0.92–2.74)	0.09	33 (22.1%)	2.22 (1.07–4.58)	< 0.05
Unemployed (*N* = 152)	130 (85.5%)			84 (55.3%)			13 (8.6%)		
Number of children									
Only child (*N* = 126)	109 (86.5%)	0.85 (0.42–1.74)	0.67	93 (73.5%)	2.16 (1.26–3.68)	< 0.01	22 (17.5%)	1.26 (0.65–2.47)	0.48
More than one child (*N* = 175)	154 (88.0%)			98 (56.0%)			24 (13.7%)		
Monthly family income									
> LKR 50,000 (N = 168)	151 (89.9%)	1.94 (0.89–4.23)	0.09	114 (67.9%)	0.95 (0.54–1.67)	0.88	34 (20.2%)	1.51 (0.70–3.26)	0.28
≤ LKR 50,000 (N = 133)	112 (84.2%)			77 (57.9%)			12 (9.0%)		

^a^The analysis was done using binary logistic regression. 301 subjects with completed socio-demographic data were included in the analysis. Adjusted odds ratios (AOR) were determined by adjusting to all other variables in the table

## Discussion

In this study, we report the patterns of screen device usage among preschool children in a suburban population of Sri Lanka. In our study population, the most used screen device was television, followed by smartphones. The pattern of usage showed a parallel distribution to the availability of devices within households. Our findings on the types of screen device usage by Sri Lankan preschool children were comparable to the usage by children from many other countries. A recent study done among pre-schoolers in India revealed that a majority were introduced to electronic devices during the second year of life, and the most common devices used were mobile phones and television [18]. Previous studies done in Canada and New Zealand also reported that preschool children's most commonly used screen device is television [9, 19]. In our study, the most frequently viewed programmes were cartoons (75.3%) and nursery rhymes (62.9%). This was consistent with previous findings from the region. A study from India revealed that 82% of television viewing preschool children watched cartoons [20].

One important observation of our study is that over 60% of the preschoolers were using screen devices for more than the recommended time limit of one hour per day. These proportions are comparable to the figures reported by preschoolers in many high-income countries [21–23]. Excessive screen time has been associated with a negative impact on language development, cognitive development, social interactions, sleep, learning, behaviour, and non-communicable diseases [1]. Therefore, it is likely that the majority of our preschool kids are at risk of developing these health hazards.

Another important observation is that over 50% of children have commenced using or viewing a screen device before completing their second birthday. The data from the USA are more alarming and shows that 92% of infants under one year have already been exposed to mobile media devices [24].

Over 75% of the informants in our study perceived that screen devices are helpful for the child's learning. This was similar to the previous findings, which reported that 50% of parents thought screens are helping their child's education [25]. Despite this belief, three-quarters of the informants in the current study had taken steps to limit their children's screen time. This is comparable to the findings of Hiniker and others, who reported that over 90% of parents had attempted to limit children's screen time [26]. The most commonly used strategies were encouraging alternate activities and limit setting, which were similar to approaches described in previous studies [27].

Our study demonstrated that the higher education level of the father is significantly associated with screen time exceeding recommended one hour per day. This contrasts with the previous report from Australia, which reported less screen time with increasing parental education level [28]. We believe that socio-cultural differences in study populations would have been the reason for this disparity. The high education level of the father in Sri Lanka strongly correlates with the income of the household and the affordability of electronic devices.

Furthermore, previous studies demonstrated significant associations between the age of the child and the screen media usage. Also, the number of children in the family had a significant impact on smartphone use. However, they did not show any significant associations with the sex of the child, maternal education, parental employment, parental age or family income. A recent systemic review found a positive relationship between screen use and the child's age. However, this analysis used the data of children aged less than 36 months [29]. Park et al. demonstrated an association between the child's age, the number of siblings, and male sex with the mobile phone addiction [12]. Furthermore, a cross-sectional study conducted in Turkey using ‘seven-in-seven screen exposure questionnaire’ revealed lower maternal age, lower maternal education level, higher paternal age and home-based daycare are associated with increased risk of problematic screen exposure in preschoolers [30]

Our paper presents the data gathered before the COVID 19 pandemic. It is very likely that the use of screen devices and screen time have markedly increased among pre-schoolers as electronic devices were extensively used for educational purposes during the COVID 19 pandemic. A recent large scale survey done in several countries reported a significant increase in screen time among preschool children globally during the pandemic [31]. Another survey carried out among children aged between 3–7 years from six countries revealed an average of 50-min increase in daily screen time during the pandemic, of which approximately 40 min accounted for entertainment [10].

One important limitation of our study is that we did not gather data separately for weekdays and weekends. Although there could be differences in screen time between weekdays and weekends, the standard preschool duration in Sri Lanka is 4 h; therefore, it is expected that most preschoolers spend the remaining time at home even on weekdays. Similarly, we did not explore the purpose of screen device use in detail. But the fact that most children used television compared to laptops suggests that the likely reason for screen device use is entertainment. Nonetheless, this is the first study that evaluates the prevalence of screen time among preschool-attending children in any part of Sri Lanka. Although we conducted this study among preschool attending children, it can represent all children in this age group of this area since almost all children aged 3–5 years in Sri Lanka attend preschools [32]. Therefore, this study provides useful information on screen device usage in the pre-COVID era to make comparisons with the use of screen devices during and after the COVID 19 pandemic.

## Conclusions

In conclusion, the electronic screen devices were used by 96% of preschoolers, and over 60% used them for more than the recommended daily upper limit of one hour. Higher paternal education, maternal employment and being the only child were significantly associated with electronic device use.

## Data Availability

The datasets used and analysed during the current study are available from the corresponding author on reasonable request.
